# Breastfeeding prevalence in newborns of mothers with COVID-19: a systematic review

**DOI:** 10.1590/0034-7167-2022-0173

**Published:** 2023-07-31

**Authors:** Mariana Torreglosa Ruiz, Karoline Faria de Oliveira, Nayara Freitas Azevedo, Marina Carvalho Paschoini, Wellington Francisco Rodrigues, Carlo José Freire de Oliveira, Jacqueline Faria de Oliveira, Luciana Mara Monti Fonseca, Monika Wernet

**Affiliations:** I Universidade Federal do Triângulo Mineiro. Uberaba, Minas Gerais, Brazil; II Universidade Federal do Triângulo Mineiro, Hospital de Clínicas. Uberaba, Minas Gerais, Brazil; III Universidade de São Paulo. Ribeirão Preto, São Paulo, Brazil; IV Universidade Federal de São Carlos. São Carlos, São Paulo, Brazil

**Keywords:** Breast Feeding, Prevalence, Infant, Newborns, COVID-19, Meta-Analysis, Lactancia Materna, Prevalencia, Recién Nacido, COVID-19, Metaanálisis, Aleitamento Materno, Prevalência, Recém-Nascidos, COVID-19, Metanálise

## Abstract

**Objectives:**

to compare exclusive breastfeeding prevalence versus artificial feeding in newborns of mothers with COVID-19.

**Methods:**

a systematic review of prevalence, according to JBI. Searches in PubMe^d^®, Embase, CINAHL, LILACS and Web of Science™ databases in August 2021. Cross-sectional, longitudinal or cohort studies were selected, without language and time limitations that showed breastfeeding prevalence or that allowed calculation.

**Results:**

fifteen articles published in 2020 and 2021, cohort (60%) or cross-sectional (40%) were analyzed. The average of exclusive breastfeeding in mothers with COVID-19 was 56.76% (CI=39.90–72.88), and artificial breastfeeding, 43.23% (CI = 30.99 – 55.88), without statistically significant differences.

**Conclusions:**

despite the recommendations for maintaining breastfeeding, there was a reduction worldwide, when compared to periods prior to the pandemic. With advances in science, these rates have improved, showing the impact of evidence on practices. As limitations, study sources are cited. It is recommended to carry out new studies. PROSPERO registration CRD42021234486.

## RESUMO

**Objetivos:**

comparar as prevalências de aleitamento materno exclusivo *versus* aleitamento artificial em recém-nascidos de mães com COVID-19.

**Métodos:**

revisão sistemática de prevalência, segundo JBI. Buscas nas bases PubMed®, Embase, CINAHL, LILACS e *Web of Science*™ em agosto de 2021. Selecionados estudos transversais, longitudinais ou coortes, sem limitação de idioma e tempo que apresentavam prevalência de aleitamento materno ou que permitissem o cálculo.

**Resultados:**

15 artigos publicados em 2020 e 2021, coortes (60%) ou transversais (40%) foram analisados. A média de aleitamento materno exclusivo em mães com COVID-19 foi 56,76% (IC=39,90–72,88), e artificial, de 43,23% (IC = 30,99 – 55,88), sem diferenças estatisticamente significantes.

**Conclusões:**

apesar das recomendações para a manutenção do aleitamento materno, houve redução mundialmente, quando comparados à períodos anteriores à pandemia. Com avanços da ciência, esses índices têm melhorado, mostrando o impacto das evidências nas práticas. Como limitações, citam-se fontes dos estudos. Recomenda-se realização de novos estudos. Registro PROSPERO CRD42021234486.

## RESUMEN

**Objetivos:**

comparar la prevalencia de lactancia materna exclusiva versus alimentación artificial en recién nacidos de madres con COVID-19.

**Métodos:**

revisión sistemática de prevalencia, según JBI. Búsquedas en las bases de datos PubMed®, Embase, CINAHL, LILACS y *Web of Science*™ en agosto de 2021. Se seleccionaron estudios transversales, longitudinales o de cohortes, sin limitaciones de idioma y tiempo que mostraran prevalencia de lactancia materna o que permitieran calcular.

**Resultados:**

se analizaron 15 artículos publicados en 2020 y 2021, de cohorte (60%) o transversal (40%). El promedio de lactancia materna exclusiva en madres con COVID-19 fue 56,76% (IC=39,90-72,88), y lactancia artificial, 43,23% (IC = 30,99 – 55,88), sin diferencias estadísticamente significativas.

**Conclusiones:**

a pesar de las recomendaciones de mantener la lactancia materna, hubo una reducción a nivel mundial, en comparación con períodos previos a la pandemia. Con los avances de la ciencia, estas tasas han mejorado, mostrando el impacto de la evidencia en las prácticas. Como limitaciones se citan las fuentes del estudio. Se recomienda realizar nuevos estudios. Registro PROSPERO CRD42021234486.

## INTRODUCTION

Since the declaration of the COVID-19 pandemic, the population and the scientific community have been concerned about SARS-CoV-2 virus implications and consequences on specific practices and populations, such as breastfeeding of newborns (NBs)^(1)^.

The World Health Organization (WHO), the Ministry of Health (MOH) and the United Nations Children’s Fund (UNICEF) recognize breastfeeding (BF) as a promoter and protector of child development, with recommendation to be practiced exclusively up to the sixth month of a child’s life and, in the mixed form (concomitant with the introduction of food), up to two years or more^(2)^. Worldwide, 80% of NBs receive breast milk at some point in their lives^(3)^, but exclusive breastfeeding (EBF) prevalence at hospital discharge is 43% and up to six months of life for children is 41%^(3)^.

Human milk itself is an appropriate food for NBs and children, it completely meets nutritional needs up to the sixth month of life^(4)^, and has exclusive immune components^(2, 5)^. Its nutritional composition and immune potential varies with gestational age, stage of lactation and health status of mother and child^(4, 5)^.

WHO data point to more than 450 million confirmed cases of SARS-CoV-2 infection and more than six million deaths worldwide due to the infection^(6)^, detected two years ago and with records of cases all over the world. Pregnant and puerperal women are classified as a risk group for infection, considered a priority in care and testing^(7)^.

In order to avoid possible post-childbirth contamination of NBs born to mothers with COVID-19, BF is recommended for mothers with mild symptoms, provided they use a surgical mask and rigorous hand hygiene^(8)^. Those with severe symptoms should be carefully assessed, but regardless, they should also be encouraged to express breast milk to start and maintain BF after the infection has resolved^(7, 9, 10)^. Expressed milk can be offered to NBs^(7, 9, 10)^.

This study considers BF prevalence among NBs born to women diagnosed with COVID-19, under the following justifications: COVID-19 is an emerging disease with a high magnitude and impact; the evidence of its effects during pregnancy and more specifically on NBs’ health are still under investigation; the benefits of BF are already enshrined in literature; early weaning rates are high. Thus, maternal infection with COVID-19 is presumed to increase weaning prevalence.

## OBJECTIVES

To compare EBF prevalence versus artificial BF in NBs of mothers with COVID-19.

## METHODS

### Study design

This is a systematic review of prevalence. According to JBI, systematic reviews of prevalence or incidence data are becoming more important as policy makers realize the usefulness of summarizing this type of information. They aim to inform and update professionals in the social and health areas, public policy makers and consumers, for decision-making in health, particularly with regard to the current health burden and its projection for the future^(11)^.

The study was registered in the International Prospective Register of Systematic Reviews (PROSPERO) database, under registration CRD42021234486, structured according to the Preferred Reporting Items for Systematic Reviews and Meta-Analyses (PRISMA) protocol^(12)^ and JBI recommendations for systematic reviews prevalence^(13)^.

The review question was based on the Condition, Context and Population (CoCoPop) strategy, establishing Co (Condition) for BF prevalence, Co (Context), for the COVID-19 pandemic, and Pop (Population), for NBs of mothers with infection by COVID-19. Based on these definitions, the review question was: what is BF prevalence in NBs of mothers with COVID-19?

### Data collection

The sources were consulted on August 2, 2021, held in the National Library of Medicine of the United States of America National Institutes of Health (PubMed®), Latin American and Caribbean Literature in Health Sciences (LILACS), Web of Science™, Excerpta Medica dataBASE (Embase) and Cumulative Index of Literature in Nursing and Related Sciences (CINAHL). The choice of databases was based on the number of indexed health articles. PubMed® is a free search engine with access to the MEDLINE database, which registers important publications of American and world literature. CINAHL is a specific database for nursing and health sciences. LILACS contains production from Latin America and the Caribbean. Embase is an important biomedical database. Web of Science™ allows the query of other databases. The objective of the diversity of bases was to contemplate the world production on the theme.

Two reviewers, both with PhDs, conducted the search independently, using controlled descriptors from Medical Subject Headings (MeSH), CINAHL Headings, Embase Emtree, and Health Sciences Descriptors (DeCS): “Breastfeeding”; “Newborns”; and “Coronavirus infections”.

The following search strategy was used in MEDLINE/PubMed®: (((“Breast Feeding”[Mesh] OR (Feeding, Breast) OR (Breastfeeding) OR (Breast Feeding, Exclusive) OR (Exclusive Breast Feeding) OR (Breastfeeding, Exclusive) OR (Exclusive Breastfeeding)) AND (“Infant, Newborn”[Mesh] OR (Infants, Newborn) OR (Newborn Infant) OR (Newborn Infants) OR (Newborns) OR (Newborn) OR (Infant) OR (Infants))) AND (“Coronavirus Infections”[Mesh] OR (Coronavirus Infection) OR (Infection, Coronavirus) OR (Infections, Coronavirus) OR (Middle East Respiratory Syndrome) OR (MERS (Middle East Respiratory Syndrome))). This strategy served as a standard for searches in other databases, with slight adaptations to the specific criteria of each database, as shown in Chart 1.

**Chart 1:** Search strategy in the consulted databases

Database	August 2021 search strategy
PubMed®/MEDLINE	((“Breast Feeding”[Mesh] OR (Feeding, Breast) OR (Breastfeeding) OR (Breast Feeding, Exclusive) OR (Exclusive Breast Feeding) OR (Breastfeeding, Exclusive) OR (Exclusive Breastfeeding)) AND (“Infant, Newborn”[Mesh] OR (Infants, Newborn) OR (Newborn Infant) OR (Newborn Infants) OR (Newborns) OR (Newborn) OR (Infant) OR (Infants))) AND (“Coronavirus Infections”[Mesh] OR (Coronavirus Infection) OR (Infection, Coronavirus) OR (Infections, Coronavirus) OR (Middle East Respiratory Syndrome) OR (MERS (Middle East Respiratory Syndrome)))
CINAHL	(Breast Feeding OR Breast Feedings OR Breastfeeding OR Breastfeedings) AND (Infant, Newborn OR Baby Newborn OR Infant OR Infants OR Newborn Infant OR Newborn Infants) AND (Coronavirus Infections OR Coronavirus Infect OR Coronavirus Infection OR Infection, Coronavirus OR Infections, Coronavirus)
Embase	(breast feeding) AND (coronavirus infection) AND (newborn)
LILACS	*(Aleitamento Materno* OR *Aleitamento* OR *Alimentação ao Peito* OR *Amamentação* OR *F01.145.407.199* OR *G07.203.650.195* OR *G07.203.650.220.500.500* OR *G07.203.650.353.199* OR *SP6.021.057.073)* AND *(Infecções por Coronavirus* OR *COVID-19* OR *Doença pelo Novo Coronavírus (2019-nCoV)* OR *Doença por Coronavírus 2019-nCoV* OR *Doença por Novo Coronavírus (2019-nCoV)* OR *Epidemia de Pneumonia por Coronavirus de Wuhan* OR *Epidemia de Pneumonia por Coronavírus de Wuhan* OR *Epidemia de Pneumonia por Coronavírus de Wuhan de 2019-2020* OR *Epidemia de Pneumonia por Coronavírus em Wuhan* OR *Epidemia de Pneumonia por Coronavírus em Wuhan de 2019-2020* OR *Epidemia de Pneumonia por Novo Coronavírus de 2019-2020* OR *Epidemia pelo Coronavírus de Wuhan* OR *Epidemia pelo Coronavírus em Wuhan* OR *Epidemia pelo Novo Coronavírus (2019-nCoV)* OR *Epidemia pelo Novo Coronavírus 2019* OR *Epidemia por 2019-nCoV* OR *Epidemia por Coronavírus de Wuhan* OR *Epidemia por Coronavírus em Wuhan* OR *Epidemia por Novo Coronavírus (2019-nCoV)* OR *Epidemia por Novo Coronavírus 2019* OR *Febre de Pneumonia por Coronavírus de Wuhan* OR *Infecção pelo Coronavírus 2019-nCoV* OR *Infecção pelo Coronavírus de Wuhan* OR *Infecção por Coronavirus 2019-nCoV* OR *Infecção por Coronavírus 2019-nCoV* OR *Infecção por Coronavírus de Wuhan* OR *Infecções por Coronavírus* OR *Pneumonia do Mercado de Frutos do Mar de Wuhan* OR *Pneumonia no Mercado de Frutos do Mar de Wuhan* OR *Pneumonia por Coronavírus de Wuhan* OR *Pneumonia por Novo Coronavírus de 2019-2020* OR *Surto de Coronavírus de Wuhan* OR *Surto de Pneumonia da China 2019-2020* OR *Surto de Pneumonia na China 2019-2020* OR *Surto pelo Coronavírus 2019-nCoV* OR *Surto pelo Coronavírus de Wuhan* OR *Surto pelo Coronavírus de Wuhan de 2019-2020* OR *Surto pelo Novo Coronavírus (2019-nCoV)* OR *Surto pelo Novo Coronavírus 2019* OR *Surto por 2019-nCoV* OR *Surto por Coronavírus 2019-nCoV* OR *Surto por Coronavírus de Wuhan* OR *Surto por Coronavírus de Wuhan de 2019-2020* OR *Surto por Novo Coronavírus (2019-nCoV)* OR *Surto por Novo Coronavírus 2019* OR *Síndrome Respiratória do Oriente Médio* OR *Síndrome Respiratória do Oriente Médio (MERS)* OR *Síndrome Respiratória do Oriente Médio (MERS-CoV)* OR *Síndrome Respiratória do Oriente Médio por Coronavírus* OR *C01.925.782.600.550.200)* AND *(Recém-nascido* OR *Criança Recém-Nascida* OR *Crianças Recém-Nascidas* OR *Lactente Recém-Nascido* OR *Lactentes Recém-Nascidos* OR *Infant OR Infants* OR *Recém-Nascido (RN)* OR *Recém-Nascidos OR M01.060.703.520)*
Web of Science™	(breastfeeding) AND (coronavirus infection) AND (newborn)

### Selection criteria

Cross-sectional, longitudinal, cohort or follow-up studies were selected, without language or time frame limitations, that presented BF prevalence or that offered data that allowed the calculation of such measure (number of infants assessed and percentage of infants breastfeeding), regardless of whether assessed as a primary outcome. Studies were included that assessed BF prevalence in NBs at hospital discharge or up to 28 days after birth and children of mothers diagnosed with COVID-19 with laboratory confirmation (PCR positive) at the time of childbirth.

Articles with secondary data (reviews), editorials, expert opinions, letters to the editor or comments on articles, case studies (only case reported), guidelines, research protocols and consensus were excluded. The level of evidence was not considered an exclusion criterion, as this is a new topic.

**Figure 1:**
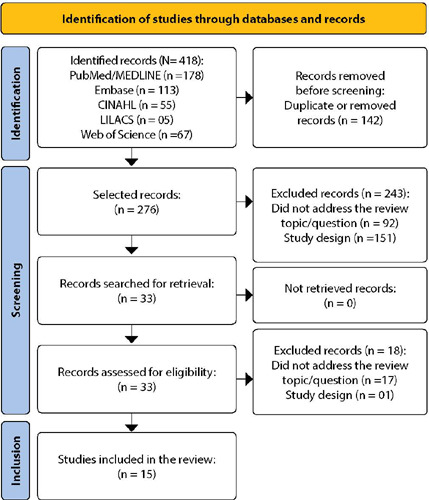
PRISMA 2020 flowchart for new systematic reviews that only included database and registry searches

Thus, 418 articles were identified in the five consulted databases. The PRISMA methodology was adopted^(12)^ and is shown in Figure 1. Study selection was carried out independently by two researchers, and disagreements were resolved by consensus. Article analysis was carried out, in a first step, with the reading of title and abstract, followed by reading in full for the final selection of articles. The order of analyzed databases was PubMed®, Embase, CINAHL, LILACS and Web of Science™. The order of exclusions followed the criteria: duplicate articles; study design inappropriate for the question - secondary data (reviews); editorials; expert opinions; letters to the editor or comments on articles; case studies (only case reported); guidelines, research protocols and consensus; and those who did not respond to the review question. Full texts were also selected in a paired and independent way.

### Data analysis and treatment

In the first step, duplicate records were removed (n= 142). Initially, the choice of articles was based on analysis of titles and abstracts. At this stage, 243 articles were excluded, as 92 did not address the theme of BF or did not make it possible to determine its prevalence and 151 did not have an adequate design for inclusion, of which, 67 were review studies on COVID-19 and maternal and child health. Then, 33 articles were read in full in an exhaustive way. One study was a review and the other 17 were excluded for the following reasons: they did not respond to the review response; lack of laboratory evidence of COVID-19; mixed samples with pregnant women without the disease; and infant assessment time (greater than 28 days). Thus, 15 studies were included in the review. The sequence of sources analyzed in the databases was PubMed®, Embase, CINAHL, LILACS and Web of Science™.

The JBI^(13)^ appraisal tools were used to assess methodological quality and risk of bias of included studies. The assessment was carried out independently by two researchers with a doctoral degree. By consensus, the group of researchers determined the cut-off point for classifying articles: as high risk of bias (score less than 50%); as moderate risk of bias (scores between 50% and 70%); and as low risk of bias (scores above 70%). The critical assessment instruments were selected according to the methodology used in the assessed studies.

Data were synthesized by two pairs of independent researchers. A structured instrument was used to extract data from the studies, following the JBI guidelines^(13)^, which included article identification, country, study setting or context, participant characteristics, groups, measured outcomes and description of main results, when cohort studies were included. For prevalence studies, article identification, country, year/period of data collection, participant characteristics, conditions and methods of measurement and description of main results were assessed. The extracted information was tabulated for data synthesis, and the analysis of results was descriptive, presenting a summary of each primary study included in this review.

Data were stored in Microsoft® Excel spreadsheets, and for analysis and visual display of the data, the RStudio program was used. Proportion meta-analysis was performed for BF prevalence and artificial, as well as for subgroup assessments (according to study design and year of publication). The General Package for Meta-Analysis “meta”, version 4.9-5, was used to analyze the proportions of BF (maternal or artificial) by mothers with COVID-19, through the “meprop” command, being adjusted with the Freeman-Tukey double arcsine transformation (sm = “PFT”), and the random effect model was used for the determinations. The forest plot, or forest graph, was used to assess and represent the data. Study heterogeneity was assessed using the I^2^ test statistic from Cochran’s Q and the J number of analyzed studies.

## RESULTS

Fifteen scientific articles were included in the analysis, nine (60%) of which were published in 2020 and six (40%) in 2021, 14 (93.3%) in English and one (0.7%) in Portuguese from Portugal. Most studies were cohorts (nine – 60%), followed by cross-sectional studies (six – 40%). The information is described in Chart 2.

**Chart 2:** Characteristics of the cohort (n=09) and prevalence studies included in the review (n=06), 2021

Characteristics of cohort studies included in the review (n=09)							
Study	Country	Setting/context	Participants	Group	Measured outcomes	Main results	Risk of bias (JBI Appraisal Tools)
Dumitriu et al., 2020^(14)^	United States	Review of analysis of medical records of mothers infected with COVID-19 and infants assisted in two large teaching hospitals in New York.	101 infants born to mothers diagnosed with COVID-19.	Analysis according to maternal symptoms: asymptomatic mothers; with mild symptoms; and with severe symptoms of the infection.	EBF rate at discharge.	- EBF prevalence (total): 40.6%; - Asymptomatic mothers: 42.9% (41/101); - With mild symptoms: 40.6% (39/91); - With severe symptoms – 20% (2/10). There was no statistically significant difference between groups.	100%
Farghaly Kupferman, Castillo & Kim, 2020^(15)^	United States	Review of electronic medical records of mothers and babies assisted at a teaching hospital in New York.	79 pregnant women tested; 15 infants born to mothers diagnosed with COVID-19.	Comparative analysis between children of mothers with COVID-19 (15) and children of mothers without infection (64).	EBF rate at discharge.	- BF prevalence in infants of mothers with COVID-19 = 33.3% (n = 5); 66.7% (n = 10) with artificial feeding; - BF prevalence in infants of mothers without COVID-19 (n = 64) = 67.2% (n = 43), with 32.8% (n = 21) on artificial BF P-value = 0.016 and risk of artificial feeding were 4 times higher in mothers with COVID-19.	100%
Gabriel et al., 2020^(16)^	Spain	Review of medical records of pregnant women diagnosed with COVID-19 at the end of pregnancy and their infants assisted in 16 hospitals in Spain.	242 pregnant women with PCR or positive serological test for COVID-19, with a follow-up of 248 infants.	Analysis at different times: after childbirth; at discharge; and in the first month of life (childcare consultation).	EBF rate at discharge.	- EBF prevalence in the first hour of life (n = 248): 54.8% (n = 136) for EBF, 28.6% (n = 71) for artificial feeding; and 16.5% (n = 41) for human milk bank milk; - At discharge (n = 247): 41.7% (n = 103) for BF; 38.4% (n = 95) for mixed (BF + formula); and 19.8% (n = 49) for artificial feeding. - In the first month (n = 235): 40.4% (n = 95) for EBF; 35.7% (n = 84) for mixed; and 23.8% (n = 56) for artificial feeding. EBF rates reduced over time.	
Malhotra et al., 2021^(17)^	United States	Review of electronic medical records of dyads (mother and infant) whose mothers were diagnosed with COVID-19, assisted in 11 hospitals with maternity care in New York.	286 dyads (mothers and infants) whose mothers tested positive for COVID-19.	Analysis in 03 groups of dyads of mothers diagnosed with COVID-19: - Positive/positive (mother and infant with positive PCR); - Positive/negative (mother positive and infant negative); - Positive/not tested (mother positive and infant not tested).	EBF rate at discharge.	EBF prevalence at discharge: - Positive/positive dyads (n = 11): 82%; - Positive/negative dyads (n=245): 55%; - Positive/not tested dyads (n = 30): 70%. The mean EBF prevalence was 57%. Higher EBF rates were observed when both had a positive result.	100%
Norman et al., 2021^(18)^	Sweden	Review of prenatal care electronic records, infant birth data and notification of COVID-19 cases in pregnant women, based on national data triangulation.	Included data for 88,159 births. Of these, 2,323 cases of mothers who tested positive for COVID-19 were analyzed.	Analysis of results of mothers with and without COVID-19.	EBF rate at discharge.	BF prevalence: - Mean: 94.3% (71,245/75,556); - 5.7% (4,311/75,556) on artificial feeding; ignored data (n = 10,280); - 94.4% (n = 1,888/2,000) in infants born to mothers with COVID-19; - 5.6% (n = 112/2,000) on artificial feeding; ignored data (n = 323); - 95.1% (n =7,873/8,281) in infants with mothers without infection; - 4.9% (408/8,281) on artificial feeding; ignored data (n = 449). There were no statistical differences between groups.	100%
Oncel et al., 2020^(19)^	Turkey	Assessment of infants born to mothers with COVID-19 who were isolated in Neonatal Care Units of 34 hospitals in Turkey.	Included 125 infants of mothers with COVID-19.	Analysis in two groups: infants born to mothers with COVID-19 with positive PCR results (n = 121); infants born to mothers with COVID-19 with negative PCR results (n = 04).	EBF rate at discharge.	BF prevalence: Infants with negative PCR: 45.6% for BF (n = 9); 7.4% for BF with precautions; 37.2% (n = 45) for expressed milk; It is 55.4% for artificial feeding. Infants with positive PCR (n = 04): 100% EBF. There were no statistical differences between groups.	100%
Popofsky et al., 2020^(20)^	United States	Survey carried out in the hospital and after discharge, via telephone contact, with mothers with COVID who had their children in 03 hospitals linked to a university in New York.	160 mothers were contacted and 85 answered the questionnaire.	Comparative analysis between binomials that were separated during hospitalization and that remained in rooming-in.	BF rate at discharge.	BF prevalence at discharge: infants in accommodation: - 27.8% for EBF; 22.2% (n = 8) for EBF; and 5.6% (n=2) for expressed milk; - 72.2% for artificial feeding; 27.8% (n = 10) for artificial feeding; - 44.4% (n = 16) for mixed BF (maternal + artificial). Infants separated from mother: - 4.1% for BF; 4.1% (n = 4) for expressed milk; for 86.9% artificial feeding; - 72.6% (n = 50) for artificial feeding; and 14.3% (n=7) for mixed feeding. p<0.001 – separation increased artificial feeding rates. The COVID-19 infection also altered maternal plans to breastfeed the NB (p <0.001).	100%
Salvatore et al., 2020^(21)^	United States	Review of medical records of mothers with COVID-19 and their infants from three New York specialty hospitals.	Of the 1,481 births, 1,16 mothers tested positive for COVID-19 (8%) and gave birth to 120 infants assessed at discharge and 82 at follow-up up to 1 month.	Assessment at four different times: during hospitalization; 5 to 7 days; 14 days; and 1 month old. Compared infants with complete follow-up (all assessments) and without follow-up.	BF rate in the first week of life.	BF prevalence between the 5^th^ and 7^th^ days of life: 78% (n = 64) for EB and 22% (n = 18) for artificial feeding. In the first month, there was an increase in EBF prevalence (85%) and a reduction in artificial feeding (15%).	100%
Sánchez-Luna et al., 2021^(22)^	Spain	Analysis of real-time electronic records of cases of children of mothers with COVID-19 from 79 Spanish hospitals.	497 mothers with COVID-19 and 503 infants.	There was no comparison between groups.	BF rate at discharge.	BF prevalence: 59.5% (n=339) of BF; 48.8% (n = 245) of EBF; 10.7% (n = 94) milk from a milk bank (donation); 40.5% (n = 203) artificial feeding; 18.5% (n = 93) mixed; 21.9% (n = 110) of artificial feeding.	100%

Characteristics of prevalence studies included in the review (n = 06)						
Study	Country	Year of data collection	Participants	Focus/method	Main results	Risk of bias (JBI Appraisal Tools)
Bartick et al., 2021^(23)^	Multicenter (31 countries)	2021	357 mothers who had been infected by COVID-19 contacted, with data from 129 infants.	BF at discharge and up to the sixth month of life - online survey.	69% (n = 86) were on EBF; 31% (n = 59) were on artificial feeding; 1.6% (n=02) did not respond. Infants on artificial BF during hospitalization were less likely to initiate or maintain BF at home.	100%
Biasucci et al, 2020^(24)^	Italy	2020	135 pregnant women tested, 15 mothers with COVID-19 (PCR positive).	BF at discharge.	EBF prevalence of 86.7% (n = 13). Two infants did not receive BF due to maternal symptoms (dyspnea).	62.5%
Brito, Sousa, Sanches, Franco, Marcelino & Costa, 2021^(25)^	Portugal	2020	77 infants born to mothers diagnosed with COVID-19.	BF at discharge.	52% (n = 40) were on EBF; 48% (n = 34) were on artificial feeding, 3% (n = 02) on artificial feeding and 45% (n = 32) on mixed feeding. Of these infants, only two had an indication for the use of a complementary formula to stabilize hypoglycemia. In neonatal return at 28 days of life, there was an increase in the EBF rate to 56% (n = 43).	100%
Cojocaru et al., 2020^(26)^	United States	2020	1,989 pregnant women were tested, of which 86 (0.04%) were PCR positive. 34 infants were assessed, and the others maintained the pregnancy.	BF at discharge.	EBF prevalence of 61% (n=16), with 11 BF cautiously and 5 with expressed milk, and 39% were on artificial feeding.	50%
Ronchi et al., 2021^(27)^	Italy	2020	62 infants born to 61 mothers with COVID-19.	BF at discharge.	BF prevalence of 75% (n =46): 73% (n =45) exclusive and 2% (n =01) expressed milk. Of the 25% on artificial feeding, 5% (n = 3) only used formula and 20% (n = 13) used mixed feeding. Artificial feeding was instituted only in cases of severe maternal infection.	100%
Sola, Rodríguez, Cardetti & Dávila, 2020^(28)^	Peru	2020	86 pregnant women who tested positive for COVID-19. 78 dyads were assessed (exclusion: two neonatal deaths and two women hospitalized in critical condition in the Critical Care Unit).	BF at discharge.	BF prevalence of 37% (n = 28): 24% (n = 18) BF with respiratory precautions and 13% (n = 10) offering expressed milk. In addition, 63% (n = 50) had artificial feeding and 76% (n = 59) of dyads were separated. It is believed that it may have contributed to the increase in artificial feeding rates.	100%

The application of tools for assessing methodological quality and risk of bias from JBI Tools made it possible to identify low risk of bias (scores above 70%) in all cohort studies included, and, among cross-sectional studies, they were classified as low risk of bias. four bias studies (66.7%), moderate risk, one study (scores between 50 and 70%) and high risk (scores below 50%), one study.

The United States was the main country producing studies (six – 40%); Italy and Spain had two publications included; a multicenter study with researchers from 31 countries was added to the review; and Peru, Portugal, Sweden and Turkey had one production each. Adding up all NBs, 4,391 children of mothers with proven COVID-19 diagnosis at the time of childbirth were assessed.

**Figure 2:**
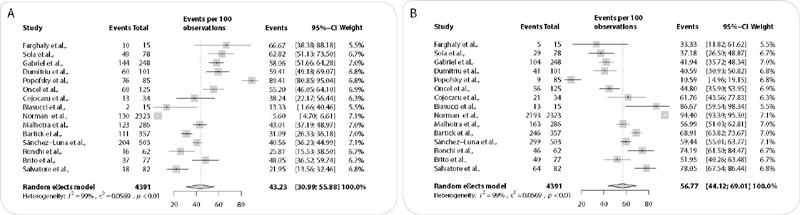
Meta-analysis to determine the influence of COVID-19 infection on breastfeeding prevalence

The mean EBF in mothers diagnosed with COVID-19 was 56.76% (CI = 39.90 - 72.88), and artificial BF, 43.23% (CI = 30.99 – 55.88). The difference was not statistically significant, however high heterogeneity is observed (I^2^ = 99%). The graphic representation is shown in Figure 2.

(A, B) forest plot of breastfeeding prevalence (A); and forest plot for artificial feeding prevalence (B).

Due to the high heterogeneity presented by the studies included, the analysis was carried out by subgroups according to study design and year of publication.

**Figure 3:**
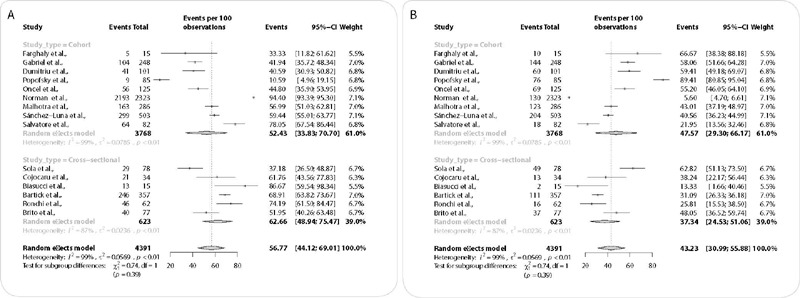
Subgroup analysis through meta-analysis to determine the influence of study design on breastfeeding prevalence in mothers with COVID-19

Figure 3 presents the analysis according to study design. In cohort studies, means of 52.43% (CI = 33.83 – 70.70) and 47.57% for artificial feeding (CI = 29.30 – 66.17) were observed. The heterogeneity observed in these studies was 99%. When analyzing cross-sectional studies, there was a reduction in heterogeneity to 87%, and the mean BF in these studies was 62.66% (CI = 48.94 – 75.47), and artificial feeding was 37.44 % (CI = 24.53 – 51.06).

(A, B) forest plot of breastfeeding prevalence according to study design – cohort or cross-sectional (A); and forest plot for artificial feeding prevalence according to study design – cohort or cross-sectional (B).

It is noteworthy that, in cohort studies, the majority was carried out through review of medical records, electronic records and online surveys. Only one study^(19)^ was carried out based on the follow-up assessment of NBs during hospitalization. Among the cross-sectional studies, the dyad was directly observed at the time of hospital discharge, except in one of them^(23)^, in which a multicentric online survey was carried out.

**Figure 4:**
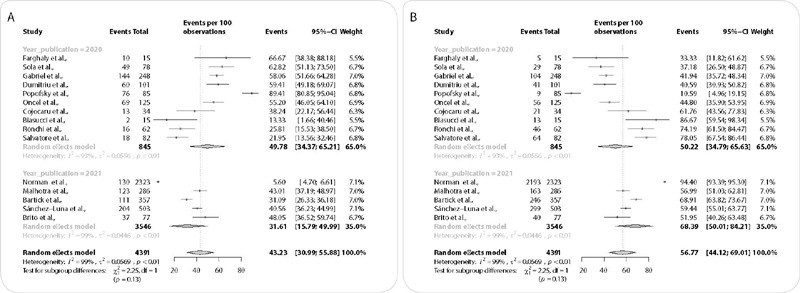
Subgroup analysis through meta-analysis to determine the influence of the year of publication of the studies on breastfeeding prevalence in mothers with COVID-19

Figure 4 presents the analysis according to the year of publication of the studies. Studies published in 2020 showed a heterogeneity of 93%, and the mean BF was 49.78% (CI = 34.37 – 65.21) and artificial feeding, 50.22% (CI = 34.79 – 65.63). Studies published in 2021 showed a heterogeneity of 99%, however there is an increase in the mean BF (68.39%) (CI = 50.01 – 84.21) and a reduction in artificial feeding (31.61%) (CI = 15.79 – 49.99).

(A, B) forest plot for breastfeeding prevalence according to year of publication – 2020 and 2021(A): and forest plot for artificial feeding prevalence according to year of publication – 2020 and 2021 (B).

## DISCUSSION

The results of this review portray worrying rates of artificial feeding among NBs of mothers with COVID-19, but when more recent publications are analyzed, a reduction in these rates is evidenced.

Due to the importance of BF, these results are cause for concern, as the Center for Disease Control (CDC) and the Royal College of Obstetricians and Gynaecologists (RCOG) do not contraindicate BF, but only indicate the use of protective measures against the spread of SARS-CoV-2^(10,17)^, due to the benefits of BF for mother and baby and lack of evidence to prove transmission through breast milk, it should be encouraged^(29, 30, 31, 32)^. It should also be mentioned that NBs born to mothers with COVID-19 are already colonized with the virus, due to previous exposure to it during pregnancy^(29, 30, 31, 32)^.

Despite the above notes, these same bodies and others agree to minimize the chances of exposure to the viral load with a contraindication for skin-to-skin contact, maintaining a distance of about two meters between the child’s crib and the mother’s bed until the infection is confirmed, frequently washing hands with soap and water and/or rubbing with 70% alcohol gel, using a disposable surgical mask and avoiding talking during feedings^(8, 10, 29, 30, 31, 32, 33, 34)^. In the presence of insecurity and fear about BF, expressing while using a surgical mask is indicated, with immediate supply of raw milk, still wearing a mask^(8, 10, 30, 31, 32, 33, 34)^.

It is important to emphasize that the evidence points to the risk of transmission through direct and intimate contact^(33)^ so that, with preventive measures and precautions, it is possible to safely establish milking and BF for asymptomatic and symptomatic mothers.

It is noteworthy that, during the pandemic period and infection duration, all nutrition options for NBs were justifiable, as it was an unknown disease; however, BF continues to be the most indicated for NBs, regardless of the infection. It reinforces the importance of guidance for mothers and families so that choices are conscious and based on scientific evidence^(35)^. These guidelines and decisions may start with COVID-19 diagnosis, but have, in the period of hospitalization after birth, a strategic moment, with direct influences on the establishment of EBF^(33)^.

A survey^(36)^ carried out in American hospitals pointed out that, from July to August 2020, of the 1,344 participating institutions, 66.9% encouraged BF with precautions; 20.1% left it as the woman’s choice, without offering support, to avoid health workers’ exposure time; 12.7% encouraged milking; and 0.2% prescribed artificial feeding (formula). It was highlighted that the reduction in BF rates can be caused by the separation/distance between mother and child, early hospital discharge (hospitalization time less than 48 hours) and the reduction of support both during hospitalization and at home^(36)^.

A study^(37)^ carried out in Italy showed a 15% reduction in BF rates, with a higher prevalence of formula use during the pandemic period. Data were statistically significant when compared to the rates presented in the year before the pandemic. Additionally, higher scores of depressive symptoms and anhedonia were observed in women who did not breastfeed their children, indicating possible long-term outcomes in maternal mental health.

A series of 22 cases described in Spain also showed that 90% of mothers infected with COVID-19 chose to breastfeed their children with precautions. However, during follow-up, it was found that, at two months, only 77% continued BF and, in all cases, no NB was infected with SARS-CoV-2^(38)^. This result suggests that longitudinal support for nursing mothers is essential to deal with the difficulties and doubts they may be experiencing^(38)^.

An online survey^(39)^ with mothers with children under one year old in England showed contradictory data. Thus, 41.8% felt that BF was protected by the pandemic, as they were able to stay at home with children, but 27% found barriers to seeking support and weaned their children early. Women with low education and of black color were more prone to early weaning during the pandemic, showing social inequalities in access to BF protection resources. According to the authors, reduced support, face-to-face contact with health professionals, mother-baby separation, confinement and reduced social support (family, friends and communities) can contribute to high weaning rates^(39)^, and the long-term impacts of increasing these rates are yet to be known.

A study^(40)^ carried out with 18 infected postpartum women in the United States pointed out that the Polymerase Chain Reaction (PCR) was positive in only one breast milk sample from a woman who was on the first day of infection. Following the analysis on the 2^nd^, 12^th^ and 41^st^ days, the PCR was negative. The positive sample, when subjected to pasteurization (heating at 62.5°C for 30 minutes and subsequent cooling to 4°C), was tested and proved negative. The authors pointed out that BF may not be a source of infection for infants and that the pasteurization process inactivates the virus^(40)^.

In a Chinese study^(41)^, breast milk samples were collected from a mother with positive PCR for COVID-19, weekly, for a period of one month. Detection of immunoglobulin G (IgG) and immunoglobulin A (IgA) was observed, with progressive increase in milk and concomitant reduction of IgG in infants’ blood until it became negative. Infants maintained negative PCR and received breast milk from birth. The authors point to the potential of immune protection of milk for NBs, suggesting that new studies be carried out to prove it^(41)^.

UNICEF data from 2018^(42)^ showed that 95% of infants worldwide received breast milk at least once in their lives, with the use of formula (artificial feeding) being more frequent in developed countries (one in five infants), compared to developing countries (one in 25). However, the same report points out that, in the same countries of origin of the reviewed studies (United States, Spain, Italy, Peru, Portugal, Sweden and Turkey), in 2018, BF rates ranged from 74.4 to 98.7 %^(42)^.

Two aspects related to the results presented must be highlighted. The first concerns the study design, as there was a predominance of data collected from medical records, online surveys or electronic systems in the cohorts carried out. The investigation of printed and electronic medical records allows knowing patients’ health conditions, but the observation relates health conditions to care and their intersections, in addition to making it possible to know the relationship of individuals with their family, with other people, with the institution, their perspectives, expectations and opinions, providing greater detail. The use of more than one method (triangulation) makes it possible to assess a reality from different perspectives and with a lower risk of bias^(43)^.

The second relates to the year of production of the publications. The reduction of artificial feeding with the advancement of science is remarkable. It is noteworthy that, after one year of the pandemic, seven aspects of progress were observed: collaboration between teams; genetic sequencing of the virus; development of different diagnostic tests; vaccine development and distribution; adjuvant treatments; greater compliance with hygiene practices by the population; and the importance of scientific research to control the pandemic^(44)^.

### Study limitations

As limitations, the predominance of data collected from medical records, online surveys or electronic systems in the cohorts carried out stands out, which can compromise the results, due to the increased risk of response bias, constituting a limitation regarding the generalization of results. Furthermore, because it is a new disease, with a rapid update of the literature and an increase in the number of cases, divergent results may appear on the subject.

### Contributions to nursing and health

Given the evidence presented, it appears that BF should be a choice of the mother and family, however, given the risks of infection and the benefits of BF, even in the presence of infection, it is strongly recommended to maintain BF with precautions. It should also be noted that the long-term impact of the increase in early weaning rates on child health and public health is unknown, requiring studies on the subject.

It is up to health professionals to offer support in this decision-making and development of children’s feeding practice. Every woman and family have the right to receive this support, which includes current and comprehensible information regarding the specificities of understanding and possibilities.

## CONCLUSIONS

EBF prevalence in NBs of mothers diagnosed with COVID-19 (56.76%) was higher than the mean for artificial feeding (43.24%). However, despite the recommendations for maintaining BF, even in the face of infection, there was a reduction in their rates, when compared to periods prior to the pandemic, which ranged from 74.4 to 98.7% in the producing countries cited in this review.

More recent studies point to a reduction in artificial feeding rates, showing the impact of evidence on practices. It is suggested to monitor the impact of weaning in the short and long term on the overall health of children born during the pandemic.
